# Chemical Analysis of the Essential Oils from Three Populations of *Lippia dulcis* Trevir. Grown at Different Locations in Southern Ecuador

**DOI:** 10.3390/plants13020253

**Published:** 2024-01-16

**Authors:** Leydy Nathaly Castillo, James Calva, Jorge Ramírez, Giovanni Vidari, Chabaco Armijos

**Affiliations:** 1 Departamento de Química, Universidad Técnica Particular de Loja, Loja 1101608, Ecuador; lncastillo@utpl.edu.ec (L.N.C.); jwcalva@utpl.edu.ec (J.C.); jyramirez@utpl.edu.ec (J.R.); 2 Department of Medical Analysis, Faculty of Applied Science, Tishk International University, Erbil 44001, Iraq; vidari@unipv.it

**Keywords:** *Lippia dulcis*, Verbeneaceae, essential oil, chemical composition, hernandulcin, GC-MS

## Abstract

In this investigation, we have analyzed for the first time the essential oils (EOs) isolated by steam distillation of the leaves and flowers of *Lippia dulcis* Trevir., grown at three different locations in southern Ecuador: the Catacocha canton (Ca), the Vilcabamba parish (Vi), and the Chuquiribamba parish (Ch). Around 98.5% of the oils’ constituents were identified by Gas Chromatography-Mass Spectrometry (GC-MS) and Gas Chromatography-Flame Ionization Detector (GC-FID) analysis using a DB-5ms capillary column. Sesquiterpene hydrocarbons were predominant in the EOs (79.77, 78.22, and 76.51%, respectively). The most representative constituents of the sample from the Ca canton were β-cedrene (16.75%), δ-selinene (11.04%), and β-cubebene (12.09%), while the sample from the Vi parish was characterized by the abundant presence of β-cedrene (17.9%), δ-selinene (12.52%), and bicyclogermacrene (11.34%). β-Cedrene (18.89%), δ-selinene (11.78%), and δ-cadinene (11.07%) were the main constituents of the essential oil (EO) from the Ch parish. The likely occurrence of low amounts of thermolabile hernandulcin in the volatile oils was indicated by the presence of the fragmentation products 6-methyl-5-hepten-2-one and 3-methyl-2-ciclohexen-1-one. In summary, the study gave us a clue to the variability of *Lippia dulcis* chemotypes depending on the collection sites.

## 1. Introduction

The plant *Lippia dulcis* Trevir., known under different vernacular names, such as the Spanish *orozuz* (“sweet grass”), is a medicinal plant belonging to the family of *Verbenaceae* Jaume St.-Hill) [1]. According to a recent classification, this family includes 32 genera and 800 species of perennial herbs, shrubs, and subshrubs of ornamental and medicinal value [2,3]. Phylogenetic studies [4,5] have shown that numerous genera traditionally classified as *Verbenaceae* belong instead to *Lamiaceae*. The plants are native to the subtropical regions of warm and semi-warm climates in Africa, Mexico, and Central America [6]. However, the flora of this taxon has become cosmopolitan thanks to the introduction of 26 genera and 180 species in North and South America, especially in Argentina and Brazil.

The Ecuadorian biodiversity of the family *Verbenaceae* is represented by 141 species, including 70 endemic ones, 22 genera, and 23 endemic taxons. They are distributed in the foothills along the “Cordillera de Los Andes”, from the Pacific coast to the páramo ecosystems. Thus, seven species are found in coastal forests, six on the islands of Galápagos, and two in the Amazon region. Due to the wide distribution, only one species (*L. salicifolia* Andersson) is protected by the National System of Protected Areas, while the remaining ones are classified as threatened. However, the names of these species are not available online [7].

Since ancient times, most verbenas, including *Lippia* species, have been known worldwide for their traditional use in the treatment of gastrointestinal disorders and respiratory problems [3]. Some species have also attributed antimalarial, antiviral, sedative, hypotensive, anti-inflammatory, and cytostatic activities. These properties are due to the presence of bioactive phytochemicals with important pharmacological effects, such as phenolic compounds and essential oils [8,9,10,11]. According to literature [10,12], 49 species of the family *Verbenaceae* grown in Ecuador are traditionally used for one or more healing purposes. Moreover, many species of *Verbenaceae* are of economic importance in agronomy and are utilized as ornamental plants and seasoning for food preservation [5].

According to different authors, the genus *Lippia* includes from 140 [2] to 200 [3] species widely distributed from the southern United States to the South and Central America regions and Tropical Africa territories [10], with the highest concentration of species in Brazil and Paraguay [13].

The genus includes aromatic herbs, shrubs, and small trees with white flowers, opposite or triple leaves, and entire or serrated with multiple buds. About the inflorescence, *Lippia* shows a zygomorphic flower, tubular calyx, and pentamerous bilabiate corolla with exalbuminate seeds [6].

*L. dulcis* is a perennial aromatic herb with leaves and flowers of intense sweetness [14,15]. Its synonyms are *Phyla scaberrima* (Juss. ex Pers.) Moldenke, *Zappania scaberrima* Juss. ex Pers., *Phyla dulcis* (Trevir.) Moldenke, *L. asperifolia* Rchb., and *L. dulcis* var. *mexicana* Wehmer [16]. The distribution of this plant in the New World extends from México, Panamá, Puerto Rico, Cuba, and other Caribbean and Central American countries to Venezuela and Colombia [15]. In Ecuador, the plant is distributed in the provinces of Loja, Azuay, Pichincha, and Zamora Chinchipe [12]. *Orozuz* uses started in the traditional medicine of the Aztecs/Mexico, who used the plant, under the Nahuatl name *Tzopelic xihuitl* (“sweet herb”), to treat bronchitis, coughs, and colds. Decoctions or infusions of the leaves and flowers are traditionally used as a topical lotion for respiratory diseases, in the treatment of diarrhea, dysentery, and indigestion, as well as an emmenagogue and a remedy to treat menstrual disorders [8,10,14]. Bactericidal, antispasmodic, antimalarial, sedative, analgesic, anti-inflammatory, antipyretic, diuretic, hypotensive, antifertility, abortifacient, and antiviral properties are attributed to the leaves and flowers of *L. dulcis* in many Latin American countries [8,10,14].

Despite the wide uses in folk medicines, it should be noted that the employment of *L. dulcis* as a low-cariogenic sweetening agent and as an additive to food and formulations of pharmaceutical products and oral hygiene products [17] is often hampered by the presence in the plant of bitter compounds, especially the toxic camphor, and other constituents with a pungent taste [15,17].

**Figure 1:**
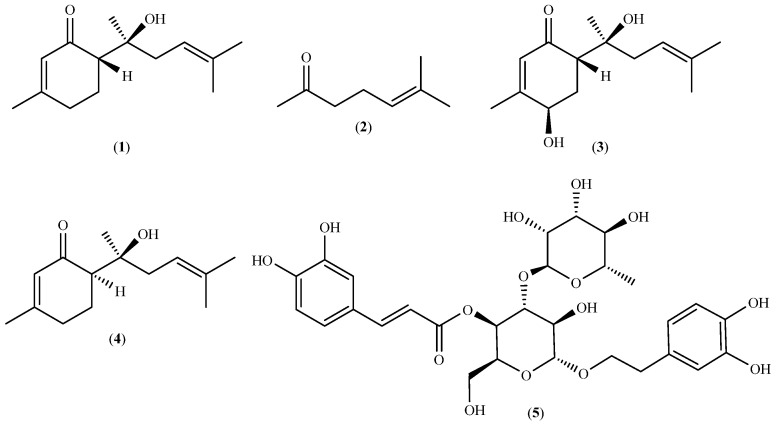
Structures of the main compounds isolated from extracts of *Lippia dulcis*.

An intensely sweet bisabolane sesquiterpene named (6*S*,1’*S*)-(+)-hernandulcin

(IUPAC name: (6*S*)-6-[(2*S*)-2-hydroxy-6-methylhept-5-en-2-yl]-3-methylcyclohex-2-en-1-one) (structure **1** in Figure 1) was first identified in an extract of dried leaves and flowers of *L. dulcis* collected in Mexico, from which it was isolated in 0.004% yield (*w*/*w*) [14,17]. The sweetness of the compound was estimated by a taste panel to be 1000 times greater than that of sucrose on a molar basis [17]. (+)-Hernandulcin was also isolated in 0.154% yield (*w*/*w*), together with 6-methyl-5-hepten-2-one (**2** in Figure 1), the sweet (+)-4β-hydroxyhernandulcin (**3** in Figure 1), and (6*R*,1′*S*)-(−)-epihernandulcin (**4** in Figure 1), which was devoid of sweetness, from a petroleum ether extract of leaves and flowers of *L. dulcis* collected in Panama, while verbascoside (**5** in Figure 1) was isolated from a methanol extract of air-dried flowers [18]. In another study, it was found that the yield of hernandulcin (**1**) in a supercritical CO_2_ extract of air-dried leaves and flowers collected in Brazil was higher for the plant harvested in the summer than in the winter [19]. The yield of hernandulcin, identified by HPLC and the GC-MS spectra of thermal dissociation products, 6-methyl-5-hepten-2-one (2) and 3-methyl-2-cyclohexen-1-one, was also higher in the summer sample [19].

The essential oil of *L. dulcis*, at the dose of 100 μg/mL, exhibited anti-histaminergic and anti-cholinergic properties [20], whereas 400 mg/kg of the ethanol extract produced significant inhibition of carrageenan-induced paw oedemas and reduced the weight of cotton pellet-induced granuloma; moreover, the topical application of 0.5 mg/ear of the ethanol extract inhibited the oedema induced with 12-*O*-tetradecanoylphorbol acetate (TPA) by 49.13% [21]. Finally, recent molecular docking studies demonstrated that some sesquiterpenes identified among the volatile compounds of *L. dulcis* from the Colombian Pacific showed very high affinity against the crystallized structures of two proteins, Mpro and PLpro, associated with Severe Acute Respiratory Syndrome COronaVirus 2 (SARS-CoV-2) [22].

The EOs of *Lippia* species contain limonene, citral, carvacrol, β-myrcene, *p*-cymene, camphor, linalool, α-pinene, β-caryophyllene, carvone, and thymol as the main chemical components [10,11]. Some examples include carvone and limonene (group I), as in the EO of *L. alba*; citral and β-caryophyllene found in *L. citriodora* (group II); and carvacrol, thymol, and *p*-cymene present in *L. origanoides* and *L. micromera* (group III). Group IV is characterized by an abundant presence of *p*-cymene and small amounts of carvacrol and thymol, as in the *L. origanoides* EO [11]. A remarkable characteristic of *Verbenaceae* EOs, including those isolated from *Lippia* species, is the variable chemical composition that determines the existence of many chemotypes, especially for species growing in South American countries [11]. The composition of *L. alba* EO well illustrates such variability [11]. In fact, the citral, carvone, and limonene chemotypes were found in different regions of Colombia, while the linalool chemotype was identified in Uruguay. On the other hand, *L. alba* growing in Argentina presents five chemotypes, namely, the linalool chemotype (up to 91%), the citral chemotype (up to 76% with two subtypes, i.e., myrcene and limonene), the piperitone chemotype (up to 37%), the lippione chemotype (up to 50%), and the dihydrocarvone chemotype [11]. The composition of *L. dulcis* oils also varied significantly with the geographic origin, especially as regards the content of camphor and hernandulcin (**1**) [23], which has suggested the existence of at least two chemotypes [24]. For example, traces of camphor were found in the volatile fractions isolated from plant specimens collected in Brazil and Puerto Rico, whereas high amounts (from 21.2 to 53.2%) were determined in the volatile oil isolated from Mexican samples [15,23]. On the other hand, variable amounts of camphor (from 0.02 to 32.6%) were detected in volatile oils from the seeds of *L. dulcis* collected in Panama [23]. Compadre et al. identified 86% of non-oxygenated and oxygenated monoterpenoids and 13% of sesquiterpenes in the volatile oil steam distilled from a dried and milled sample of the plant collected in Mexico [15]. The presence of 6-methyl-5-hepten-2-one (**2**) (0.51%), likely formed by thermal degradation of hernandulcin (**1**), was detected in this sample [15]. Instead, hydrodistillation of leaves of *L. dulcis* harvested in July in Colombia afforded an oil richer in the sesquiterpene fraction (96.1%) than in the monoterpenoid one (3.9%) [24]. Among the 32 identified sesquiterpenes, α-copaene (18.0%), β-caryophyllene (17.8%), and δ-cadinene (14.7%) were the major compounds, while bisabolane hernandulcin and 6-methyl-5-hepten-2-one formed only 1.1% and 2.4%, respectively, of the oil. Camphor was not detected in the oil [24]. A high percentage of sesquiterpenoids (79.5%). was also determined in the volatile oil from the plant collected in Puerto Rico, in which the sesquiterpenoids hernandulcin (**1**) (36%) and its epimer *epi*-hernandulcin (**4**) (22%) were the main components [23]. The oil contained, if any, undetectable amounts of camphor (< 0.01%). Adams et al. found that the content of the volatile oil from a Brazil sample was high in 6-methyl-5-hepten-2-one (10.5%), α-copaene (8.6%), (*E*)-caryophyllene (10.6%), bicyclogermacrene (6.6%), δ-cadinene (7.2%), *epi*-α-bisabolol (6.5%), and hernandulcin (8.8%). In addition, the oil contained β-cedrene and α-calacorene [25]. Instead, the oil from a Mexican *L. dulcis* sample different from that analyzed by Compadre [15] was high in camphene (12.7%), limonene (4.6%), camphor (33.9%), α-copaene (4.0%), (*E*)-caryophyllene (6.0%), and hernandulcin (5.9%) [25]. In addition, it contained alkanes and fatty acids (docosane, tricosane, tetracosane, pentacosane; linoleic and octadecanoic acids) that were not found in the oil from the Brazil sample [25]. Noteworthy, precipitation, temperature, and plant age influenced, to some extent, the content and the chemical composition of the EO isolated from leaves of *L. dulcis* collected in Amazonia [26]. The EO content varied from 0.1 to 0.5%, whereas the composition did not change significantly with the seasons. The main constituents were *epi*-α-bisabolol and hernandulcin (**1**), whose concentration reached its peak in the dry season, characterized by mild temperatures and the juvenile stage of plant growth. Instead, the highest amounts of *epi*-α-bisabolol were detected in the hottest and wettest months [26].

**Figure 2:**
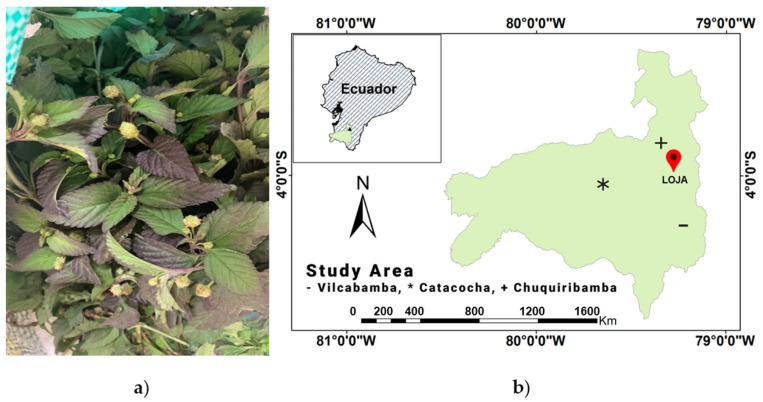
(**a**) *L. dulcis* (**b**) Collection locations of *L. dulcis* in the Loja province in southern Ecuador.

The chemical composition of the volatile oil from *L. dulcis* Trevir. grown in Ecuador (Figure 2a) has not been investigated so far. In this inaugural study that we intend to extend to the entire country, we describe the components of the oil steam distilled from the leaves and flowers of *L. dulcis* grown in the Loja province in southern Ecuador. To investigate the variability of the oil composition, three samples were obtained at three different locations near the town of Loja: the Catacocha canton (Ca), the Vilcabamba parish (Vi), and the Chuquiribamba parish (Ch) (Figure 2b). In the folk medicine of local communities, infusions of the flowers and roots of *L. dulcis* are widely used for stopping menstrual bleeding and treating stomach inflammation [12].

Moreover, we were highly interested in the determination of the hernandulcin and camphor contents in view of the possible application of the plant as a sweetener. In this regard, the taste of the three samples of *L. dulcis* examined in this work was characterized by the presence of sesquiterpenes of the bisabolane type, such as hernandulcin.

## 2. Results

### 2.1. Physical Properties of the EOs

The three EOs were obtained in yields ranging from 0.08 to 0.20%, depending on the plant samples. They had a pale-yellow color, a fresh aroma, and a pleasant, sweet taste. The relative density, refractive index, and optical rotation of the oils are reported in Table 1.

### 2.2. Chemical Composition of the EOs

**Figure 3:**
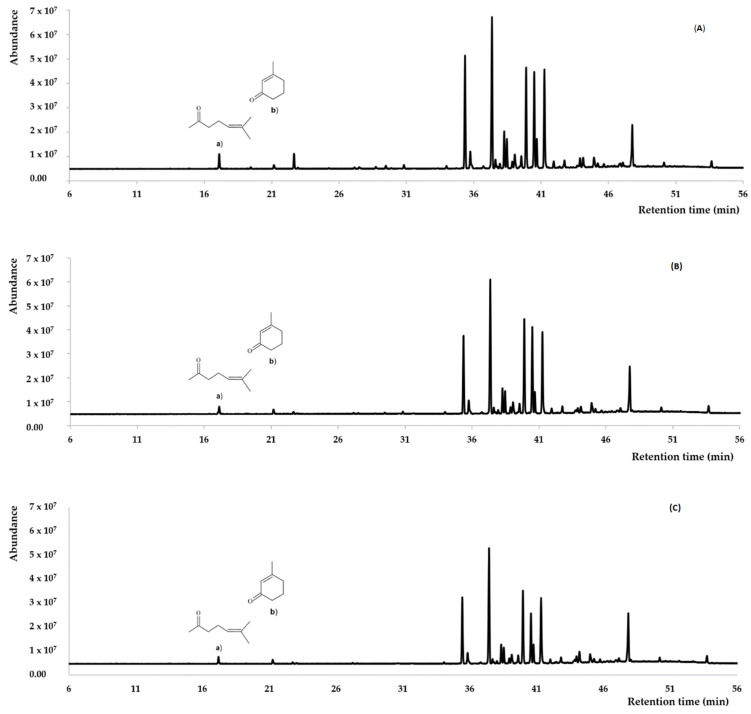
Gas chromatograms of *L. dulcis*. EOs from (**A**) Catacocha, (**B**) Vilcabamba, and (**C**) Chuquiribamba, on a DB-5ms column, thermal dissociation products of hernandulcin: a) 6-methyl-5-hepten-2-one, b) 3-methyl-2-cyclohexen-1-one.

A total of 76 compounds with percentages ˃ 0.05% were identified in the three EOs steam distilled from fresh leaves and flowers of *L. dulcis*. They accounted for 98.23, 97.39, and 99.89%, respectively, of the Ca, Vi, and Ch EOs analyzed on a DB-5ms (dimethylsilicone phase with 5% phenyl groups) capillary column (Figure 3). The content (%) of each identified oil component was calculated as the % of the area of the corresponding peak in the Gas Chromatography-Flame Ionization Detector (GC-FID) chromatogram compared to the sum of the areas of all identified peaks. No correction factor was applied. The values of percentage and standard deviation were the means of three determinations. Each EOs component was identified by comparing the calculated linear retention index (LRI) on the DB-5ms column and/or the GC-MS spectrum with the PubChem (nih.gov) database (https://pubchem.ncbi.nlm.nih.gov, accessed on 1 September 2023) and the references [27,28,29]. The LRIs were calculated according to the method of Van Den Dool and Kratz [30], using a mixture of *n*-alkanes (C_9_–C_25_) injected under the same chromatographic conditions as the EOs. The qualitative and quantitative compositions of the oils are reported in Table 2.

Indeed, the analysis of the three EOs was tentatively also carried out on a capillary column containing a polar polyethylene glycol stationary phase (INNOWax). We observed, however, that the peaks were generally more overlapped than on the DB-5ms column; moreover, we found that the LRIs reported in the literature for the EO constituents are often greatly different, making compound identification rather uncertain. Therefore, the data from the GC analysis on the polar column have not been considered.

The components occurring in the three EOs with percentages >3% on the DB-5ms column are listed in Table 3, while their chemical structures are depicted in Figure 3. Only the relative stereochemistry is shown.

## 3. Discussion

Transparent greenish EOs were steam distilled from fresh samples of *L. dulcis* collected at three different locations in southern Ecuador. The EO from Catacocha showed the highest isolated yield (0.20%, *w*/*w*). However, it should be optimized according to the season and extraction technique. In fact, this value must be compared with the 0.6% and 1.7–3.4% yields obtained by hydrodistillation and supercritical fluid extraction, respectively, of dried *L. dulcis* collected in Brazil [19]. On the other hand, the relative density and the refractive index of the oils (Table 1) resembled those of *L. alba* collected in Brazil [31]. Around 98.5% of the chemical constituents of *L. dulcis* EOs from the three Ecuadorian locations were identified. The GC-FID and GC-MS analyses on the DB-5ms column (Table 2) indicated that the EOs consisted mainly of sesquiterpene hydrocarbons (79.77, 78.22, and 76.51%, respectively), followed by oxygenated sesquiterpenoids (12.69, 15.67, and 20.38%, respectively). Oxygenated monoterpenoids (2.18, 0.74, and 0.27%, respectively) and other compounds (3.59, 2.76, and 2.73%, respectively) occurred in the EOs as minor components. Noteworthy, monoterpene hydrocarbons were not detected in the three EOs, and the percentages of only nine compounds were ˃3% (Table 3).

**Figure 4:**
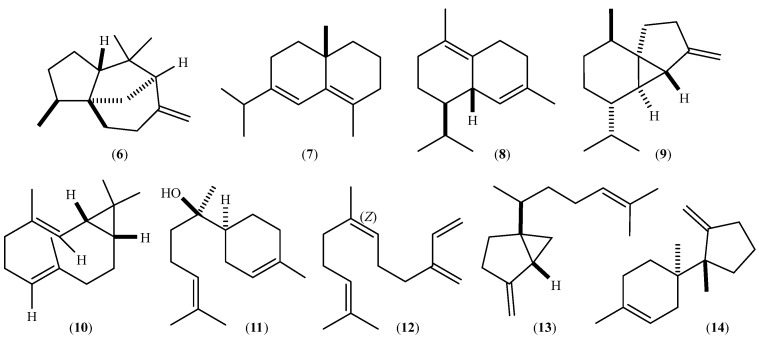
Chemical structures of the main components of the EOs of *Lippia dulcis* from Ecuador.

The profiles of the three oils were similar qualitatively (Figure 3), but the quantitative compositions were significantly different. Thus, the five most abundant components were the same (Table 3). β-Cedrene (**6** in Figure 4) and β-cubebene (**9**) predominated in the Ca oil, while β-cedrene (**6**) and δ-selinene (**7**) were the most abundant constituents of the Vi and Ch oils (Table 3). On the other hand, the Ch oil contained a higher amount of α-bisabolol (**11**) than the other two oils, while *(Z)-*β-farnesene (**12**) was not abundant (Table 3). Minor components also characterize each oil: 1,8-cineole, salvial-4(14)-en-1-one, methyl hexadecanoate, 1-octadecanol, and methyl octadecanoate were only identified in the Ca EO; γ-muurolene, γ-patchoulene, *cis*-cadinene ether, *trans*-cadinene ether, silphiperfol-5-en-3-ol A, (*E*)-tridec-2-en-1-yl acetate, and α-eudesmol acetate were only detected in the Vi EO; percentages ˃ 0.05% of isoledene, cumacrene, γ-amorphene, *n*-pentadecane, 10-*epi*-cubebol, dodecanoic acid, *trans*-sesquisabinene hydrate, *cis*-dihydro-mayurone, widdrol, germacra-4(15),5,10(14)-triene-1-α-ol, khusilal, 2,3-dihydrofarnesol, melaleucol, hinesol acetate, and (*E*)-α-atlantone were only identified in the Ch EO.

It should be noted that camphor and hernandulcin (**1**) were not detected in any of the three volatile oils. Even the products formed by thermal degradation of hernandulcin [15], 6-methyl-5-hepten-2-one (**2**), and 3-methyl-2-ciclohexen-1-one were found in low amounts.

In striking contrast to the EOs isolated from other *Lippia* species, in which monoterpenoids predominated [11], and to the oils from *L. dulcis* collected in Mexico, which were rich in camphor [15,25], the high content of sesquiterpene hydrocarbons characterized the EOs from Ecuadorian specimens. This feature also characterized the EOs from *L. dulcis* collected in Puerto Rico [23], Colombia [22,24], and Brazil [25], which, however, contained different sesquiterpenes as the most abundant components, while hernandulcin (**1**) and 6-mehyl-5-hepten-2-one occurred in the volatile oils in various amounts (see the Introduction above). These findings, in particular the apparently minimal accumulation of hernandulcin and the absence of camphor in the volatile oils, indicate that *Lippia dulcis* grown in southern Ecuador may constitute a new chemotype of this species.

## 4. Materials and Methods

### 4.1. Collection of Plant Material

Fresh leaves and flowers of *Lippia dulcis* Trevir. were purchased from April to June 2022 from medicinal plant vendors at three different locations in the province of Loja, southern Ecuador: (i) at the Cangonama parish of the Catacocha canton, at an altitude of 1460 m a.s.l. (GPS coordinates: 3°58′0″ S, 79°42′0″ W); (ii) at the San Francisco neighborhood of the Vilcabamba parish, at 1700 m a.s.l. (GPS coordinates: 4°15′0″ S and 79°15′0″ W); (iii) at the Carmelo sector of the Chuquiribamba parish, at 2600 m a.s.l. (GPS coordinates: 03°75′8″ S, 79°15′00″ W). The UTPL botanist Jorge Armijos identified the botanical material, and vouchers of the three plant samples have been deposited at the Herbarium of the Universidad Técnica Particular de Loja (HUTPL) with the codes HUTPL 14610–14612.

### 4.2. Essential Oil Isolation

The three EOs of *Lippia dulcis* Trevir. were obtained from fresh leaves and flowers (approximately 2.15 kg of plant from each location) by steam distillation for 3 h using Clevenger-type equipment. After decantation, each supernatant oily layer was separated from the condensed aqueous phase using a pipette; subsequently, each oil (3.5, 0.85, and 1.22 mL of Ca, Vi, and Ch, respectively) was dried over anhydrous Na_2_SO_4_, filtered, and stored in an amber vial at 4 °C until use. The procedure was performed in triplicate.

### 4.3. Physical Properties

The relative density of the EOs from *L. dulcis* was determined according to the international standard AFNOR NF T75-111 (ISO 279:1998) [32] guidelines. The refractive index was determined using an ABBE refractometer, manufactured by Boeco (Hamburg-Germany), according to the international standard AFNOR NF 75-112 (ISO 280:1998) [33] procedure. The specific optical rotation $[{a]}_{D}^{20}$ was measured according to the ISO 592:1998 [34] procedure using an automatic polarimeter Hanon P 810, manufactured by Hanon Advanced Technology Group (Jinan-China). For each physical property, the mean value and the SD were determined from three replicates performed at 20 °C.

### 4.4. Chemical Composition of Essential Oils

#### 4.4.1. Gas Chromatography Coupled to Mass Spectrometry (GC-MS)

The *L. dulcis* EOs were qualitatively analyzed by GC-MS using an Agilent Technologies Chromatograph 6890N coupled to an Agilent mass spectrometer, the 5973 Inert series (Santa Clara, CA, USA), which operated in electron impact mode at 70 eV. The mass spectrometer was operated in SCAN mode (range 35–350 *m*/*z*), had a scan rate of 0.2 scan/s, and was controlled by MSD-Chemstation D.01.00SP1.

The reported results of the EOs analysis were obtained on a DB-5ms (5% phenyl-polymethylsiloxane; 30 m × 0.25 mm × 0.25 µm, Agilent Technologies; J; W Scientific, Folsom, CA, USA) capillary column; helium (1.00 mL/min) was used as the carrier gas. The injection system operated in split mode (40:1) at 220 °C. The program for the GC oven temperature was as follows: initial temperature of 60 °C, held for 5 min, followed by a ramp of 3 °C/min until 250 °C, which was finally held for 10 min. Each EO was diluted with CH_2_Cl_2_ (1:100 *v*/*v*), and 1 μL of the solution was injected into each analysis.

To calculate LRIs, a homologous series of *n*-alkanes C_9_–C_25_ (C_9_, BHD purity 99%, and C_10_–C_25_, Fluka purity 99%) were injected after each EO under identical chromatographic conditions.

#### 4.4.2. Gas Chromatography Coupled to a Flame Ionization Detector (GC-FID)

The EOs were quantitatively analyzed by GC-FID on an Agilent Technologies 6890N series gas chromatograph coupled to a flame ionization detector (FID), using the DB-5ms column mentioned in Section 4.4.1. The analytical gas-chromatographic conditions were the same as those used for the GC-MS analysis.

## 5. Conclusions

The chemical composition and the physical properties of the steam distilled EOs from *L. dulcis* collected at three locations in southern Ecuador (Catacocha, Vilcabamba, and Chuquiribamba) were determined for the first time. The profiles of the three oils markedly differed from the EOs of *L. dulcis* collected in other Central and South American countries, confirming that the chemical composition of *L. dulcis* volatile oils can vary significantly with the geographic origin of the plant. Instead, the EOs isolated from the three samples collected in Ecuador were similar qualitatively, and this finding may depend on the proximity of the three locations, even if the altitudes varied from 1460 to 2600 m a.s.l. The minimal concentration of hernandulcin (**1**) in the EOs must be confirmed by collecting the plant in other locations of Ecuador, in different periods of the year, at different stages of growth, and using different extraction and analysis conditions [19]. Moreover, the absence of camphor, if confirmed, is promising for the sustainable use of *L. dulcis* as a natural, non-cariogenic sweetener.

## Figures and Tables

**Table 1:** Physical properties of EOs from *Lippia dulcis* Trevir. collected in southern Ecuador.

Collection Location	Yield % (*v/w*)	Relative Density g/mL	$\mathrm{Refractive}\;\mathrm{Index}\;n_{D}^{20}$	$\mathrm{Optical}\;\mathrm{Rotation}\;[{a]}_{D}^{20}$
Catacocha	0.20	0.91 ± 0.02	1.4895	+11.38
Vilcabamba	0.08	0.86 ± 0.02	1.4993	+9.70
Chuquiribamba	0.17	0.84 ± 0.02	1.5005	+18.14

**Table 2:** Chemical composition of *L. dulcis* essential oil from fresh plant samples collected at Catacocha, Vilcabamba, and Chuquiribamba.

Compound	RT (min)	LRI_calc_^a^	LRI_lit_^b^	Collection Location		
				Catacocha	Vilcabamba	Chuquiribamba
				% ± SD	% ± SD	% ± SD
6-Methyl-5-hepten-2-one	17.1	994	986	1.58 ± 0.12	0.97 ± 0.19	1.06 ± 0.00
1,8-Cineole	19.45	1038	1032	0.15 ± 0.03	-	-
3-Methyl-2-cyclohexen-1-one	21.17	1070	1039	0.48 ± 0.1	0.71 ± 0.17	0.74 ± 0.12
Unknown A, MW 168 *	22.49	1209	-	0.15 ± 0.01	0.11 ± 0.00	-
*trans*-Sabinene hydrate	22.66	1107	1098	1.52 ± 0.16	0.28 ± 0.00	0.27 ± 0.01
*n*-Nonanal	22.95	1114	1103	0.12 ± 0.01	-	0.14 ± 0.00
Isoamyl tiglate	27.14	1202	1196	0.14 ± 0.01	0.44 ± 0.00	0.15 ± 0.01
Neral	28.73	1250	1242	0.18 ± 0.00	0.18 ± 0.02	-
Geranial	29.47	1280	1270	0.33 ± 0.01	0.28 ± 0.01	-
α-Longipinene	33.98	1351	1352	0.28 ± 0.01	0.23 ± 0.00	0.23 ± 0.00
Isoledene	35.16	1372	1377	-	-	0.06 ± 0.01
γ-Amorphene	35.24	1495	1495	-	-	0.05 ± 0.01
β-Cubebene	35.35	1389	1387	12.09 ± 0.34	9.96 ± 0.06	10.51 ± 0.22
β-Bourbonene	35.74	1381	1384	2.00 ± 0.02	2.13 ± 0.01	2.04 ± 0.00
Sibirene	35.87	1393	1394	0.34 ± 0.01	0.03 ± 0.00	0.28 ± 0.01
β-Funebrene	36.71	1413	1415	0.32 ± 0.00	0.27 ± 0.00	-
β-Cedrene	37.35	1427	1422	16.75 ± 0.02	17.90 ± 0.01	18.89 ± 0.20
α-Guaiene	37.61	1438	1440	0.92 ± 0.01	0.78 ±0.00	0.70 ± 0.02
Aromadendrene	37.74	1446	1441	0.21 ± 0.03	0.21 ± 0.00	0.37 ± 0.01
Allo-aromadendrene	37.94	1452	1458	0.47 ± 0.00	0.44 ± 0.00	2.42 ± 0.00
(*E*)-β-Farnesene	38.26	1457	1456	3.65 ± 0.24	3.11 ± 0.01	0.20 ± 0.01
Sesquisabinene	38.45	1459	1457	3.21 ± 0.02	2.79 ± 0.02	2.85 ± 0.07
Cumacrene ^c^	38.95	1461	1490	-	-	0.84 ± 0.01
β-Acoradiene	39.05	1468	1469	1.82 ± 0.01	0.83 ± 0.01	1.65 ± 0.02
7-*epi*-1,2-Dehydrosesquicineole	39.41	1475	1473	0.16 ± 0.12	1.76 ± 0.02	0.14 ± 0.00
γ-Muurolene	39.51	1477	1477	-	0.17 ± 0.01	-
Germacrene D	39.53	1483	1481	1.42 ± 0.05	1.39 ± 0.01	1.37 ± 0.02
δ-Selinene	39.89	1490	1492	11.04 ± 0.7	12.52 ± 0.05	11.78 ± 0.3
γ-Patchoulene ^c^	40.25	1498	1474	-	0.14 ± 0.02	-
*n*-Pentadecane	40.3	1498	1500	-	-	0.11 ± 0.00
Bicyclogermacrene	40.48	1505	1504	10.77 ± 1.24	11.34 ± 0.02	8.14 ± 0.24
β-Bazzanene ^c^	40.68	1515	1528	3.14 ± 0.12	2.77 ± 0.02	2.94 ± 0.09
δ-Cadinene	41.25	1527	1524	11.25 ± 0.73	11.14 ± 0.1	11.07 ± 0.32
γ-Cuprenene	41.52	1533	1532	0.09 ± 0.02	0.07 ± 0.01	0.12 ± 0.00
10-*epi*-Cubebol	41.85	1539	1535	-	-	0.60 ± 0.00
*cis*-Dracunculifoliol	41.94	1545	1542	0.73 ± 0.04	-	0.41 ± 0.04
*cis*-Sesquisabinene hydrate	42.25	1543	1542	0.16 ± 0.14	0.64 ± 0.00	0.18 ± 0.00
*cis*-Cadinene ether	42.37	1548	1552	-	0.06 ± 0.01	-
*trans*-Cadinene ether	42.4	1554	1557	-	0.24 ± 0.00	-
Unknown B, MW 204 *	42.52	1561	-	0.76 ± 0.02	-	-
Unknown C, MW220 *	42.64	1564	-	0.12 ± 0.02	-	-
Silphiperfol-5-en-3-ol A	42.65	1564	1547	-	0.06 ± 0.00	-
(*E*)-Nerolidol	42.74	1569	1561	0.78 ± 0.12	0.88 ± 0.02	0.94 ± 0.02
Dodecanoic acid	42.91	1572	1569	-	-	0.06 ± 0.00
*trans*-Sesquisabinene hydrate	43.24	1578	1580	-	-	0.07 ± 0.00
Unknown D, MW 268 *	43.71	1588	-	-	0.20 ± 0.00	-
Dihydromayurone	43.91	1588	1595	-	-	1.07 ± 0.04
Spathulenol	43.89	1591	1578	1.06 ± 0.11	0.36 ± 0.00	-
Widdrol	44.14	1593	1597	-	-	1.55 ± 0.07
Caryophyllene oxide ^c^	44.12	1596	1581	1.26 ± 0.08	0.64 ± 0.01	-
Salvial-4(14)-en-1-one	44.57	1600	1595	0.06 ± 0.12	-	-
Unknown E, MW 222 *	44.58	1601	-	-	0.95 ± 0.00	0.05 ± 0.01
Viridiflorol ^c^	44.61	1620	1592	-	0.05 ± 0.00	0.04 ± 0.01
β-Biotol	44.92	1626	1616	1.04 ± 0.2	1.21 ± 0.02	1.28 ± 0.03
Unknown F, MW 220 *	45.20	1628	-	-	0.50 ± 0.02	-
α-Acorenol	45.21	1632	1630	0.52 ± 0.03	0.28 ± 0.04	0.43 ± 0.01
4(15),5,10(14)-Germacratrien-1α-ol ^c^	45.37	1637	1686	-	-	0.1 ± 0.01
Aromadendrene epoxide	45.66	1641	1639	0.40 ± 0.04	0.11 ± 0.01	0.66 ± 0.01
14-Hydroxy-9-*epi*-(*E*)-caryophyllene ^c^	45.96	1648	1664	0.17 ± 0.02	0.07 ± 0.01	0.51 ± 0.02
Khusilal	45.97	1650	1648	-	-	0.14 ± 0.05
α-Muurolol	46.24	1662	1646	0.24 ± 0.02	0.14 ± 0.01	0.32 ± 0.07
Neointermedeol	46.44	1672	1660	0.24 ± 0.03	0.18 ± 0.01	0.19 ± 0.01
*epi*-α-Bisabolol	46.88	1676	1683	0.41 ± 0.03	0.19 ± 0.01	0.25 ± 0.01
(*Z*)-γ-Atlantone ^c^	47.08	1682	1698	0.52 ± 0.05	0.48 ± 0.02	0.67 ± 0.01
2,3-Dihydrofarnesol	47.24	1685	1688	-	-	0.13 ± 0.00
Unknown G, MW 220 *	47.50	1699	-	0.64 ± 0.02	0.66 ± 0.01	0.51 ± 0.01
α-Bisabolol	47.77	1701	1690	4.52 ± 0.46	6.14 ± 0.06	8.68 ± 0.05
(*Z*)-*epi*-β-Santalol	47.96	1706	1704	-	0.73 ± 0.00	0.11 ± 0.01
(*Z*)-Apritone ^c^	48.46	1712	1689	-	0.29 ± 0.00	-
Drim-8-en-7-one ^c^	48.93	1807	1792	0.10 ± 0.04	-	0.13 ± 0.01
(*E*)-Tridec-*2*-en-1-yl acetate	48.94	1725	1703	-	0.07 ± 0.01	-
Flourensadiol	49.33	1869	1864	0.06 ± 0.28	1.00 ± 0.20	1.30 ± 0.02
Melaleucol ^c^	49.41	1729	1706	-	-	0.07 ± 0.01
Methyl linoleate	50.13	2102	2097	0.41 ± 0.76	0.04 ± 0.07	-
(*E*)-2-Hexyl-cinnamaldehyde	50.14	1745	1748	-	0.53 ± 0.01	0.42 ± 0.01
β-Bisabolenal	50.58	1767	1768	0.26 ± 0.03	0.06 ± 0.01	0.12 ± 0.01
Hinesol acetate	50.92	1778	1798	-	-	0.09 ± 0.05
Methyl hexadecanoate	51.58	1934	1927	0.08 ± 0.29	-	-
(*E*)-α-Atlantone	51.65	1786	1777	-	-	0.2 ± 0.01
α-Eudesmol acetate	51.79	1791	1795	-	0.1 ± 0.00	-
Cyclopentadecanolide	52.83	1844	1834	-	-	0.05 ± 0.01
1-Octadecanol	53.66	2109	2089	0.72 ± 1.63	-	-
Methyl octadecanoate	54.05	2135	2128	0.06 ± 0.21	-	-
Monoterpene hydrocarbons (%)				0.00	0.00	0.00
Oxygenated monoterpenoids (%)				2.18	0.74	0.27
Sesquiterpene hydrocarbons (%)				79.77	78.22	76.51
Oxygenated sesquiterpenoids (%)				12.69	15.67	20.38
Other identified compounds (%)				3.59	2.76	2.73
Total identified compounds (%)				98.23	97.39	99.89

RT = retention time; ^a^ LRIcalc = calculated linear retention index; ^b^ LRI_lit_ = linear retention index taken from literature; ^c^ tentatively identified by the MS spectrum; %, percentage; SD, standard deviation. Both values were calculated as the mean of three determinations; * EIMS data for unknown compounds. Unknown A: *m*/*z* (%) 168 (~0.1, M^+^), 156 (1), 123 (1), 95 (25), 83 (43), 82 (30), 70 (1), 69 (53), 67 (22), 55(31), 53 (7), 43 (10), 41 (55), 40 (100); Unknown B: *m*/*z* (%) 204 (15, M^+^), 200 (6), 183 (1), 161 (1), 158 (2), 157 (35), 156 (7), 143 (3), 142 (22), 128 (3), 115 (6), 105 (2), 55 (4), 53 (2), 44 (4), 41 (6), 40 (100); Unknown C: *m*/*z* (%) 220 (~0.1, M^+^), 200 (6), 183 (1), 161 (1), 158 (2), 157 (35), 156 (7), 143 (3), 142 (22), 128 (3), 115 (6), 105 (2), 55 (4), 53 (2), 44 (4), 41 (6), 40 (100); Unknown D: *m*/*z* (%) 268 (~0.1, M^+^), 207 (1), 165 (1), 105 (1), 123 (2), 111 (2), 95 (6), 83 (6), 81 (8), 71 (5), 69 (8), 67 (9), 57 (6), 55 (14), 54 (3), 43 (9), 41 (11), 40 (100); Unknown E: *m*/*z* (%) 222 (~0.1, M^+^), 204 (15), 161 (11), 148 (5), 139 (6), 134 (9), 133 (8), 121 (24), 105 (17), 93 (48), 91 (15), 69 (100), 55 (31), 43 (97); Unknown F: *m*/*z* (%) 220 (~0.1, M^+^), 205 (2), 187 (3), 177 (6), 163 (5), 149 (9), 135 (12), 121 (26), 109 (41), 95 (53), 93 (75), 79 (100), 69 (68), 55 (56), 53(28), 44 (18), 43(98), 41(89); Unknown G: *m*/*z* (%) 220 (~0.1, M^+^), 205 (2), 187 (3), 177 (6), 161 (8), 165 (1), 159 (5), 147 (6), 133 (9), 120 (14), 119 (18), 109 (41), 107 (37), 82 (45), 69 (100), 55 (56), 41 (89).

**Table 3:** Main components of the EOs of *L. dulcis*.

Compound	Collection Location		
	Catacocha (%)	Vilcabamba (%)	Chuquiribamba (%)
β-Cedrene (**6**)	16.75 ± 0.02	17.90 ± 0.01	18.89 ± 0.2
δ-Selinene (**7**)	11.04 ± 0.66	12.52 ± 0.05	11.78 ± 0.3
δ-Cadinene (**8**)	11.25 ± 0.73	11.14 ± 0.1	11.07 ± 0.32
β-Cubebene (**9**)	12.09 ± 0.34	9.96 ± 0.06	10.51 ± 0.22
Bicyclogermacrene (**10**)	10.77 ± 1.24	11.34 ± 0.02	8.14 ± 0.24
α-Bisabolol (**11**)	4.52 ± 0.46	6.14 ± 0.06	8.68 ± 0.05
(*Z*)-β-Farnesene (**12**)	3.65 ± 0.24	3.11 ± 0.01	0.20 ± 0.01
Sesquisabinene (**13**)	3.21 ± 0.02	2.79 ± 0.02	2.85 ± 0.07
β-Bazzanene (**14**)	3.14 ± 0.12	2.77 ± 0.02	2.94 ± 0.09

## Data Availability

All data presented in this study are available in the article.
